# Frontal Metabolite Concentration Deficits in Opiate Dependence Relate to Substance Use, Cognition, and Self-Regulation

**DOI:** 10.4172/2155-6105.1000286

**Published:** 2016-07-15

**Authors:** Donna E Murray, Timothy C Durazzo, Thomas P Schmidt, Christoph Abé, Joseph Guydish, Dieter J Meyerhoff

**Affiliations:** 1 Center for Imaging of Neurodegenerative Diseases (CIND), San Francisco VA Medical Center, San Francisco, CA, USA; 2 Department of Radiology and Biomedical Imaging, University of California San Francisco, San Francisco, CA, USA; 3 Department of Psychiatry and Behavioural Sciences, Stanford University School of Medicine, Stanford, CA, USA; 4 Mental Illness Research Mental Illness Research and Education Clinical Centers; Sierra-Pacific War Related Illness and Injury Study Center, VA Palo Alto Health Care System, Palo Alto CA, USA; 5 Department of Clinical Neuroscience, Osher Center, Karolinska Institutet, Nobelsväg 9, 17177 Stockholm, Sweden; 6 Philip R. Lee Institute for Health Policy Studies, University of California San Francisco, San Francisco, CA, USA

**Keywords:** Alcoholism, Brain, Cognition, Opiate, Proton magnetic resonance spectroscopy, Smoking

## Abstract

**Objective:**

Proton magnetic resonance spectroscopy (1H MRS) in opiate dependence showed abnormalities in neuronal viability and glutamate concentration in the anterior cingulate cortex (ACC). Metabolite levels in dorsolateral prefrontal cortex (DLPFC) or orbitofrontal cortex (OFC) and their neuropsychological correlates have not been investigated in opiate dependence.

**Methods:**

Single-volume proton MRS at 4 Tesla and neuropsychological testing were conducted in 21 opiate-dependent individuals (OD) on buprenorphine maintenance therapy. Results were compared to 28 controls (CON) and 35 alcohol-dependent individuals (ALC), commonly investigated treatment-seekers providing context for OD evaluation. Metabolite concentrations were measured from ACC, DLPFC, OFC and parieto-occipital cortical (POC) regions.

**Results:**

Compared to CON, OD had lower concentrations of N-acetylaspartate (NAA), glutamate (Glu), creatine +phosphocreatine (Cr) and myo-Inositol (mI) in the DLPFC and lower NAA, Cr, and mI in the ACC. OD, ALC, and CON were equivalent on metabolite levels in the POC and γ-aminobutyric acid (GABA) concentration did not differ between groups in any region. In OD, prefrontal metabolite deficits in ACC Glu as well as DLPFC NAA and choline containing metabolites (Cho) correlated with poorer working memory, executive and visuospatial functioning; metabolite deficits in DLPFC Glu and ACC GABA and Cr correlated with substance use measures. In the OFC of OD, Glu and choline-containing metabolites were elevated and lower Cr concentration related to higher nonplanning impulsivity. Compared to 3 week abstinent ALC, OD had significant DLPFC metabolite deficits.

**Conclusion:**

The anterior frontal metabolite profile of OD differed significantly from that of CON and ALC. The frontal lobe metabolite abnormalities in OD and their neuropsychological correlates may play a role in treatment outcome and could be explored as specific targets for improved OD treatment.

## Introduction

The misuse of opiates is a serious problem worldwide, is increasing in young adults [1–3], and has substantial individual and societal consequences. In 2014 in the United States alone, approximately 1.9 million people had an opiate use disorder, including 586,000 heroin users [2]. Neuroimaging in opiate dependence indicates both altered brain structure, particularly in the anterior cingulate cortex (ACC; [4–7]), and brain function involving dorsolateral prefrontal cortex (DLPFC) and ACC [8,9]. Magnetic resonance spectroscopy (1H MRS) allows the non-invasive quantitation of brain metabolites that provide information on the neurophysiologic integrity of brain tissue [10]. The few 1H MRS studies in opiate dependence to date revealed lower concentration of N-acetylaspartate (NAA), a marker of neuronal integrity, in the medial frontal cortex, including the ACC [11–13], as well as lower glutamate (Glu), a primary excitatory neurotransmitter, or glutamate+glutamine concentration in some [11,13,14] but not all studies [15]. The discrepant MRS findings may relate to differences among study participants regarding the prevalence and severity of comorbid substance use (i.e., alcohol, tobacco, illicit drugs), the type, dose and duration of replacement therapy for heroin users (buprenorphine, methadone), and/or participant age.

The ACC, DLPFC and orbitofrontal cortex (OFC) are important components of the brain reward/executive oversight system, a neural network critically involved in the development and maintenance of addictive disorders [16,17]. Structural brain imaging in opiate dependence revealed generally lower gray matter volume or density in (pre)frontal regions [5–7,9,18], including the DLPFC [9,19], with thinner frontal cortices related to longer duration of opiate misuse [4]. Functional MR imaging showed that the DLPFC, OFC and ACC are involved in decision making [20–22], and in opiate dependent individuals, lower task-based fMRI activity in the ACC [8] related to compromised behavioural control of cognitive interference [8,11]. Furthermore, smaller frontal gray matter volume in opiate dependence related to higher impulsivity on the Barratt Impulsivity Scale (BIS-11; [19,23]). Correspondingly, opiate dependence is associated with cognitive deficits [24–28], primarily in executive functioning and self-regulation (impulsivity, decision-making, risk taking [19,29]). Thus, the neuroimaging literature in opiate dependence suggests altered frontal brain structure as well as compromised neuronal integrity and glutamatergic metabolism. Few if any studies however investigated their relationships to opioid and other substance use behaviour or cognition. Further research into specific regional brain effects and their potential cognitive and behavioural correlates may inform better targeted treatment of individuals with opioid use disorders.

We measured in opiate dependent individuals’ metabolite concentrations from the ACC and previously unexplored DLPFC and OFC and related them to quantitative measures of neurocognition, self-regulation, and substance use. Specifically, we compared opiate dependent individuals (OD) on buprenorphine maintenance to controls (CON). We also included another control group, a substance-dependent ‘control’ group of 3 week abstinent alcohol dependent individuals (ALC), a commonly investigated treatment-seeking group to differentiate opiate dependence from not only control individuals but also individuals with a substance dependence (here, alcohol dependence). Our primary hypotheses were that: (1) OD have lower NAA and Glu concentrations than CON in ACC, DLPFC, and OFC, (2) these frontal cortical NAA and Glu deficits are associated with the level of opiate use and cigarette-smoking severity, (3) the frontal NAA and Glu deficits in OD relate to higher impulsivity, poorer executive function, and lower decision making skills, and (4) OD have more pronounced metabolite concentration deficits than ALC. The results of this study will contribute to a better understanding of the neurobiology and neuropsychology in OD, helping to identify novel targets for the treatment of opiate dependence.

## Materials and Method

### Participant characterization

All participants provided informed consent according to the Declaration of Helsinki and underwent procedures approved by the University of California, San Francisco and San Francisco VA Medical Center (Federalwide Assurance (FWA) 00000068). Twenty-one chronic cigarette smoking OD, stable on buprenorphine maintenance therapy for at least 3 months, met DSM-IV criteria for dependence on opiates; they were allowed to meet DSM-IV criteria for current abuse or dependence on cocaine, amphetamines, and/or cannabis, but dependence on alcohol or benzodiazepines was exclusionary. OD was part of a buprenorphine treatment program focusing on smoking cessation and they were studied before smoking cessation. For group comparisons of metabolite concentrations specifically in the ACC, DLPFC, and POC and when correlated with neuropsychological variables, there were data from thirty-five cigarette smoking ALC recruited from local treatment programs of the VA and Kaiser Permanente and 28 cigarette smoking CON recruited from the community. The ALC group met DSM-IV criteria for alcohol dependence and was abstinent from alcohol (not tobacco) for 21 ± 11 days at time of study. For group comparisons of metabolite concentrations in the OFC and when correlated with neuropsychological variables (the OFC VOI only), smokers and non-smokers were included in the ALC and CON groups: 21 ALC (9 nonsmokers, 12 smokers) and 19 CON (14 non-smokers, 5 smokers) due to a lack of sufficient data in smokers. All participants were studied with structural MRI, 1H MRS, and neuropsychological testing, all were fluent in English and they were allowed to smoke ad libitum before assessment and during breaks. Table 1 contains demographics, tobacco and alcohol use variables, mood measures, and laboratory variables for the three groups.

Further exclusion criteria for ALC and CON are described elsewhere [30]. In brief, ALC and CON participants were excluded for neurological disorders (e.g. seizures, neurodegenerative disorder, traumatic brain injury with loss of consciousness >5 min), psychiatric disorders (e.g. history of schizophrenia spectrum, bipolar and panic disorders, posttraumatic stress disorder), and medical and vascular risk factors (e.g. endocrine diseases, chronic obstructive pulmonary disease, type-1 diabetes, myocardial infarction, cerebrovascular accident, migraine headaches), known to affect neurobiology or cognition as well as for MRI contraindications. In OD and ALC, hepatitis C, type-2 diabetes, hypertension, unipolar mood disorder, or generalized anxiety disorders were not exclusionary due to their high prevalence in addiction [31–35]. Six OD, 4 ALC and 1 CON had hepatitis C (by self-report and medical chart review), while 4 OD and 13 ALC had medically-controlled hypertension.

All OD were on buprenorphine maintenance therapy averaging 15 ± 9 mg/day. Table 2 depicts their recent and lifetime substance use histories. Overall, OD as a group were all cigarette smokers (by design) and had comorbid stimulant and marijuana use over lifetime, which they reduced during the year before study. Only a few OD individuals had drug use within the last 30 days: 3 used opiates and/or cocaine but only 1 used opiates for 20 days, 1 other OD used amphetamines daily, and about one-third of the sample used marijuana. The majority of OD individuals were moderate alcohol drinkers over their lifetime, but they reduced their alcohol consumption during the last year before study; only 3 had consumed alcohol on more than 10 days within the last 30 days. The ALC group for the ACC, DLPFC, and POC VOI analyses were cigarette smokers abstinent from alcohol for about 3 weeks and used other drugs occasionally (5 ALC used marijuana and 1 ALC used cocaine within the last 30 days). Thus, the ALC group for the majority of the analyses (3 of 4 VOIs) and the entire OD group were cigarette-smoking treatment seekers, abstinent from their main drug of abuse for several weeks and they had similarly low levels of drug use within the last month before study.

### Clinical, neurocognitive and behavioural assessment

OD and ALC completed the Structured Clinical Interview for DSM-IV Axis I disorders Patient Edition, v2.0 [36], CON were administered the corresponding screening module. The clinical and neurocognitive assessments of ALC and CON are detailed elsewhere [30]. In all groups, alcohol consumption was estimated with the lifetime drinking history interview [37,38], nicotine dependence was assessed with the Fagerstrom Tolerance Test for Nicotine Dependence [39], and lifetime substance use history (other than alcohol) was assessed with an in-house questionnaire [40]. All participants completed the Beck Depression Inventory (BDI; [41]) and the State-Trait Anxiety Inventory (STAI; [42]).

A neurocognitive battery assessed the major domains affected by opioid and alcohol use disorders and Z-scores were calculated based on corresponding normative data. Cognitive domains were formed from specific neurocognitive tasks (see [30] for details and Table 3). The cognitive domain scores in ALC and CON were calculated according to the shortened neurocognitive battery of tests administered to the OD group and therefore, the constituent measures for cognitive domains in this study are different from our previous publications. All participants completed self-regulation measures, which included the BIS to assess self-reported impulsivity, the Balloon Analogue Risk Task (BART; [43]) to assess risk taking, and the Iowa Gambling Task (IGT; [44]) to assess decision making. Laboratory tests within 2–3 days of the MR scan evaluated the nutritional status and alcohol-related or other hepatocellular injury in OD and ALC. See Table 1 for laboratory variables, cognitive domain and self-regulation measures for the three groups.

### Magnetic resonance methods

**Figure 1:**
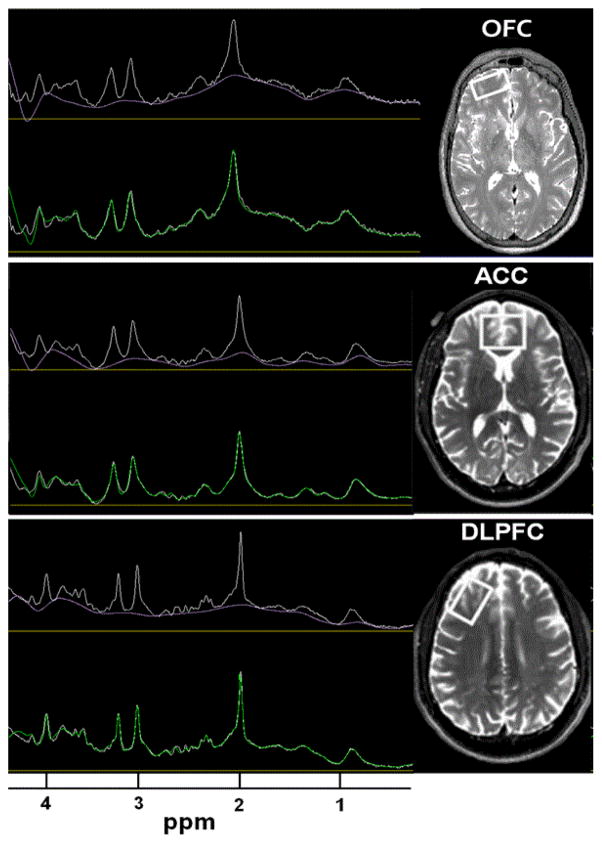
OFC compared to ACC and DLPFC VOI STEAM spectra shown as fitted after DC-correction and apodization. For all VOIs, the experimental spectra are represented in white and the fitted baseline is displayed in purple. The green line overlying the white line in the bottom spectra for each VOI represent the convergence of the summed spectral fits for each metabolite resonance.

MR data were acquired on a 4 T Bruker MedSpec system with a Siemens Trio console (Siemens, Erlangen, Germany) using an 8- channel transmits-receive head coil. 3D sagittal T-1-weighted and 2D axial T2-weighted images were acquired using Magnetization Prepared Rapid Gradient imaging (TR/TE/TI=2300/3/950 ms, 7º flip angle, 1 × 1 × 1 mm^3^ resolution) and turbo spin-echo (TR/TE=8400/70 ms, 150º flip angle, 0.9 × 0.9 × 3 mm^3^ resolution) sequences, respectively. NAA, creatine+phosphocreatine (Cr), choline containing metabolites (Cho), myo-Inositol (mI) and Glu signals in MRS volumes-of-interest (VOIs) were acquired with a Stimulated Echo Acquisition Mode (STEAM) sequence (TR/TE/TM=2000/12/10 ms, 90º flip angle, 2000 Hz spectral bandwidth, 2.5 min) ([45]) and placed over the ACC (35 × 25 × 20 mm^3^), right DLPFC (20 × 40 × 20 mm^3^), right OFC (40 × 20 × 10 mm^3^) at the base of the inferior prefrontal cortex, and medial parieto-occipital region (POC; 40 × 20 × 20 mm^3^) to maximize the corresponding cortical gray matter (GM) content. See Figure 1 for VOI placements and example MR spectra. γ-aminobutyric acid (GABA) signals from ACC, DLPFC and POC were acquired from the exact same VOIs with a modified J-editing sequence (MEGA PRESS: TR/TE=2000/71, 90º flip angle, 2000 Hz spectral bandwidth, 12.5 min) [46]). STEAM and GABA spectra were not always acquired from all VOIs in all participants and the numbers of VOI-specific spectra analysed are shown in Table 4. The corresponding structural MR images were segmented into GM, white matter (WM), and cerebrospinal fluid (CSF; [47]) to estimate tissue fraction and CSF contributions to each VOI for calculation of metabolite concentrations in institutional units (i.u). Quantitated metabolite concentrations were corrected for CSF contribution and scaled to the water level from the corresponding VOI (Table 4).

For full methods details see [48]. Twelve percent of CON and 47% of ALC participants of the current study were included in our previous reports on metabolite concentrations in individuals with alcohol and poly-substance dependence [48,49].

### Statistical analyses

Univariate analyses of covariance (ANCOVA) tested for group differences on demographic and clinical variables. All statistical analyses were performed with SPSS version 22. Separate ANCOVAs were performed for the four VOIs and each metabolite, followed by planned pairwise comparisons to test for group differences in metabolite concentrations between OD, ALC and CON. Given the participants’ wide age range (23–60 years) and as age correlates with metabolite concentrations (e.g., [50]), age was used as a covariate in group comparisons. As GM, WM, and CSF contributions to the VOIs affect brain metabolite levels [51] and as tissue content in ACC and OFC VOIs differed between groups (see Table 4), we included these variables as predictors in the ANCOVAs.

Each a priori hypothesis was tested with an alpha level of 0.05. In pairwise group comparisons of metabolite levels without a specific a priori hypothesis, we used corrected alpha levels to account for the multiplicity of metabolites in each VOI via a modified Bonferroni procedure [52], which yielded adjusted alpha levels for each VOI separately by using the number of metabolites under investigation and their average inter-correlation coefficients (ACC: r=0.26; DLPFC: r=0.42; OFC: r=0.62; POC: r=0.44). The adjusted alpha levels for statistical significance were p=0.018 for ACC, 0.022 for DLPFC, 0.038 for OFC, and 0.020 for POC. OFC spectra often did not have a well-defined mI resonance (overlap with residual water) and therefore, OFC mI was not analysed. Effect sizes were calculated via Cohen’s d [53]. Correlations between outcome measures were corrected for age (i.e., partial correlations), except for correlations with cognitive domains (based on age-adjusted normative data), and reported as Pearson coefficients.

## Results

### Participant characterization

Age and years of education did not differ between OD and CON (Table 1). ALC were equivalent on age to OD and CON, but had fewer years of education. OD had lower hemoglobin and hematocrit than both CON and ALC. There were no significant differences in blood tests of liver function (γ-GTP, Albumin, Aspartate Aminotransferase, Alanine Aminotransferase and Alkaline Phosphatase) in individuals with and without Hepatitis C within the ALC group and also within the OD group. In addition, none of the individuals with Hepatitis C were taking medications at the time of study for Hepatitis C. Furthermore, the individuals taking hypertension medication did have controlled blood pressure by self-report but blood pressure levels at time of study were not measured. Nicotine dependence scores were higher in OD than ALC; OD and CON also smoked significantly more cigarettes per day than ALC, but all groups were equivalent on cigarette smoking duration. Gender did not contribute to any group difference or correlation. See Table 1 for drinking severity measures in OD, CON and ALC.

### Group comparisons of metabolite concentrations

Significant main effects of group were observed for NAA, Cr, and mI in the ACC and for NAA, Cr, mI, and Glu in the DLPFC (Table 5). In pairwise comparisons of OD and CON, the DLPFC showed the greatest magnitude metabolite concentration differences, with effect sizes up to 1.64. Specifically, NAA, Glu, Cr, and mI were all significantly lower in OD (all p<0.01) than CON and ALC. OD also had lower NAA, Cr, and mI concentrations in the ACC (all p<0.001), while ACC Glu tended to be lower than in CON (p=0.06). GABA concentrations did not differ between OD and CON in any region. In the OFC, metabolite concentrations were not different between OD and CON, while Cho tended to be higher in OD (p=0.09). The CON group for OFC comparisons comprised both smokers and non-smokers; in previous MRS research, smoking CON revealed metabolite deficits compared to non-smoking CON in DLPFC NAA, Cr, mI and Glu [54]. Here, we found lower OFC Cho and Glu in smoking versus non-smoking CON (effect size 1.55). Correspondingly, OFC Glu and Cho were significantly higher in OD than smoking CON (effect sizes 0.6–1.4), with no group differences for OFC NAA and Cr. In contrast to frontal VOI metabolite concentrations, POC NAA, Cr, Cho, mI and Glu concentrations did not differ significantly between OD and CON. The 3 week abstinent ALC did not differ significantly from CON in DLPFC metabolite concentrations, however, ALC had NAA and Cr reductions in the ACC similar to those of OD. In the OFC, ALC (comprised of both smokers and non-smokers) had significantly higher Glu and Cho than CON (potentially driven by the smaller proportion of smokers among CON).

### Associations between metabolite concentrations and cigarette smoking measures

Cigarette smoking measures did not correlate significantly with metabolite concentrations in OD, but trends emerged: ACC NAA and Cr tended to correlate negatively with more cigarettes/day (both r>−0.39, p<0.08). In sCON, ACC Glu was negatively associated with pack-years (r=−0.41, p=0.04, statistical trend), and in sALC, FTND score and cigarettes/day was positively related to OFC mI (both r>0.70, both p<0.02).

### Associations between regional metabolite concentrations and substance use in OD

**Figure 2:**
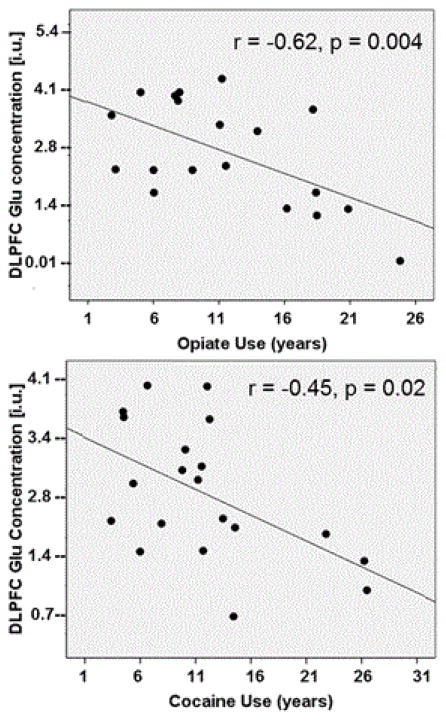
In OD, the associations of DLPFC Glu concentration (in institutional units, i.u.) with opiate and cocaine use.

Greater substance use in OD related to altered metabolite concentrations, after adjusting for age: DLPFC Glu was negatively associated with lifetime duration of opiate (r=−0.62, p=0.004) and cocaine use (r=−0.45, p=0.02) (Figure 2), whereas DLPFC NAA did not correlate with any substance use measure. ACC GABA correlated negatively with monthly opiate use in the previous year and with monthly cocaine use over lifetime (both r>−0.47, both p<0.043, trends after multiple comparison correction). In addition, ACC Cr correlated negatively with monthly marijuana use in the previous year (r=−0.54, p=0.016) and over lifetime (r=−0.47, p=0.03, statistical trend) and positively with amphetamine use in the previous month (r=0.49, p=0.03, statistical trend). Finally, POC Cr correlated negatively with longer duration of opiate use (r=−0.58, p=0.014) and mI correlated negatively with monthly opiate use in the previous year (r=−0.65, p=0.003).

### Cognitive domains and self-regulation in OD

OD had better executive functioning scores than (smoking) CON (p=0.01), but did not differ on any other cognitive domain, decision making, or risk taking measure (Table 1). Also, OD did not differ significantly from abstinent ALC on cognitive domain scores, decision making or risk-taking. In OD, working memory related negatively to lifetime years of opiate use (r=−0.53, p=0.01). OD performed in the average range of functioning across all domains based on the domain z-scores derived from normative data.

### Associations of metabolite concentrations with cognition and self-regulation measures

In OD, DLPFC NAA concentration correlated with executive function (r=0.54, p=0.024, uncorrected), and NAA and Cho correlated with visuospatial skills and global cognition (all r>0.51, all p<0.031, uncorrected). Also in CON, DLPFC NAA correlated with visuospatial skills (r=0.47, p=0.01). In OD, ACC Glu correlated with working memory (r=0.50, p=0.02); the low NAA and Glu concentrations in DLPFC and ACC did not correlate with any of our measures of self-regulation; only OFC Cr was negatively related to non-planning impulsivity (r=−0.65, p=0.021).

## Discussion

This study compared cortical metabolite concentrations, neurocognition, and self-regulation between cigarette-smoking opiate dependent individuals on buprenorphine maintenance therapy, treatment-seeking alcohol dependent smokers, and smoking controls. OD had significant metabolite alterations in markers of neuronal integrity (NAA), cell membrane turnover/synthesis (Cho), glutamate concentration (Glu), cellular bioenergetics (Cr), and astrocyte integrity (mI) in frontal lobe regions implicated in the development and maintenance of addictive disorders. OD had lower NAA, Glu, Cr and mI concentrations than CON in the DLPFC and lower NAA, Cr and mI in the ACC. The metabolite concentration deficits in OD were most pronounced in the DLPFC, were associated with various substance use measures, and correlated with worse performance on measures of global cognition, executive and visuospatial functioning. However, OD and CON were equivalent in regional GABA concentrations, most cognitive domains, and self-regulation measures. Relative to 3 week abstinent ALC, OD had significantly lower NAA, Cr, Cho and mI concentrations in the DLPFC, with NAA and Cho deficits having cognitive ramifications.

Consistent with most previous reports [11–14], we found metabolite deficits in the ACC of OD. In addition, OD had similar deficits in NAA, Cr, and Glu concentrations in the DLPFC. This suggests reduced neuronal and astrocyte viability and cellular bioenergetics in both the ACC and DLPFC, with additional glutamatergic injury in the DLPFC. ACC Glu and also DLPFC NAA and Cho metabolite abnormalities related to poorer cognitive function, which, however, did not differ significantly from CON. Of note, GABA concentrations in ACC and DLPFC of OD were equivalent to those in smoking CON, similar to findings in 3-week abstinent ALC versus smoking CON (this study) and 1 week abstinent ALC vs. mostly non-smoking CON [48]. However, ACC GABA reductions were reported in abstinent individuals with cocaine- [55] and polysubstance-dependence [49]. The POC and occipital region have been used as control regions in MRS studies as they are typically not altered in addiction [56,57]. This appears to be true also for OD, who showed the most pronounced metabolite deficits in anterior frontal brain regions.

We also assessed the OFC region previously not investigated in OD. The lateral OFC subserves motivation, drive, reward valuation, and aspects of social executive skills, is affected in opiate dependence [58] and other drug abuse [59], and the OFC has altered brain activity in decision making task-based fMRI studies of individuals with substance use disorders [60]. OFC metabolite concentrations did not differ between OD and CON, the latter including mostly non-smokers. However, and in contrast to DLPFC and ACC findings, OD showed elevated Glu and Cho concentrations in the OFC when compared to a subset of CON, the small group of smoking CON. Although the small group size warrants caution when interpreting results, our finding of lower OFC Cho concentration in smoking vs. non-smoking CON is consistent with lower Cho measured in frontal, midbrain and vermis regions of smoking vs. non-smoking controls [61].

In OD, lower DLPFC Glu and strong trends for lower ACC GABA correlated with greater severity and duration of opiate use. These findings are congruent with other neuroimaging studies that reported lower DLPFC GM density [9,18] and poorer functional connectivity between DLPFC and parietal regions associated with greater duration of opiate use [9]. ACC Glu and NAA were not related to opiate use, consistent with previous reports [11]. However, greater cocaine and marijuana misuse in our OD group was associated with significantly lower metabolite concentrations, commensurate with findings in other substance using/dependent populations [62–64].

Metabolite concentrations in the DLPFC and ACC of OD related to executive function, visuospatial skills, global cognition and working memory, but not to self-regulation measures. Previous 1H MRS studies in opiate dependence did not report on such relationships, but studies in marijuana-dependent and recreational ecstasy users reported relationships between altered frontal metabolite levels and impaired cognition or higher impulsivity [56,65,66]. Although previous research in opiate addicts reported neuropsychological deficits [24,25], our OD group performed in the average range across various cognitive domains and self-regulation measures. There is some evidence that buprenorphine maintenance is associated with better cognition compared to other maintenance drugs [67–70], and buprenorphine has been shown to improve brain perfusion in cocaine dependence [71,72]; correspondingly, buprenorphine may have had an effect on cognitive performance in OD in this study. Future studies on the effects of buprenorphine on brain function and cognition in OD may be useful to inform effective treatment.

Our study showed that OD on maintenance therapy had greater anterior frontal brain metabolite abnormalities than 3 week abstinent ALC, and we found previously that even 1 week abstinent ALC did not show metabolite abnormalities in the DLPFC [48]. The greater DLPFC metabolite abnormalities in OD may relate to the greater relapse rate in opiate than alcohol dependence [73], which may require differently tailored approaches for treatment of OD and ALC. Metabolite deficits in the DLPFC of OD are more reminiscent of 1H MRS results in poly-substance users [49,64], recreational cannabis users [62], and methamphetamine dependent individuals [63]. The DLPFC is critically involved in executive functions, such as working memory, cognitive flexibility, planning, inhibition, and abstract reasoning. As such, DLPFC brain metabolite abnormalities, in addition to those in ACC, may be promising targets to monitor the efficacy of cognitive behaviour therapy in OD treatment, especially as they correlate with cognition and substance use behaviour.

This study has limitations. Drug use histories were based on self-report and gender effects across groups could not be assessed due to the small number of females (21%). Menstrual cycle appears to affect brain GABA levels [74], but data on the time since last menstrual cycle was not collected. However, excluding the female participants from statistical analyses did not alter the finding of no significant GABA differences between groups. The number of analysed spectra for some comparisons was relatively small, especially those involving smoking CON with OFC and POC VOIs; therefore, these analyses need to be considered hypothesis generating rather than definitive. Further, differences to previous metabolite and neuropsychological research in OD may relate to differences in comorbid tobacco, alcohol, marijuana and stimulant abuse as pointed out previously [28]. Of note in this context is the relatively low lifetime and current alcohol use in our OD sample. An additional limitation is that the duration of buprenorphine maintenance therapy was not assessed, although OD had to be on therapy for at least 3 months. Furthermore, the results may not be generalizable to OD who are not on buprenorphine therapy. Finally, we cannot rule out the possible contributions of premorbid, developmental, and dietary/nutritional factors to the neurobiological group effects reported.

## Conclusion

Our findings of regional metabolite concentration abnormalities in the absence of neuropsychological deficits in OD are of clinical significance. They extend previous reports of ACC metabolite abnormalities in OD to DLPFC and OFC, all important components of brain circuitry relevant to relapse risk, and they include comparisons with smoking CON. While the findings are largely consistent with the broader literature on prefrontal brain deficits in substance users, they also expose differences of the frontal metabolite profile between OD and ALC, revealing metabolic abnormalities in OD more similar to those of polysubstance, cannabis and methamphetamine users and related to cognitive performance, opiate, and comorbid substance use. In efforts to facilitate endogenous neuroplasticity, these metabolite abnormalities and comorbid substance use should be explored as important targets in the treatment of opiate dependence including heroin addiction. From a methodological point-of-view and because MRS measures are related to cognition, quantitative 1H MRS may be useful for monitoring both pharmacological and cognitive behavioural therapy intended to facilitate abstinence in OD.

## Figures and Tables

**Table 1:** Demographics, alcohol and tobacco use, mood symptomatology, cognitive domains, self-regulation, and laboratory variables for OD, ALC and CON (mean ± standard deviation), NS: Non-Significant (p>0.05); FTND: Fagerstrom Tolerance Test for Nicotine Dependence; BIS-11: Barratt Impulsivity Scale version 11; BART: Balloon Analogue Risk Task; IGT: Iowa Gambling Task. Blood tests with non-significant differences between groups: Albumin, Aspartate Aminotransferase, Alanine Aminotransferase, Alkaline Phosphatase, Chloride, Cortisol, Carbon Dioxide, Glucose, Osmolality, Prealbumin, Protein, Red and White Blood Cell count and Sodium.

Variable	OD	ALC	CON	p (OD vs. CON)	p (OD vs. ALC)	p (ALC vs. CON)
Total n [male, female]	21 (13,8)	35 (29,6)	28 (24,4)			
*Demographics*						
Age [years]	41.1 ± 11.7	46.6 ± 8.8	44.2 ± 8.5	NS	0.053	NS
Education [years]	14.4 ± 1.5	13.3 ± 1.6	14.6 ± 2.0	NS	0.013	0.004
Body Mass Index	26.2 ± 5.3	25.9 ± 5.4	26.2 ± 3.4	NS	NS	NS
*Cigarette and alcohol use, mood measures*						
FTND total	4.8 ± 1.4	3.6 ± 1.5	4.5 ± 1.8	NS	0.009	0.044
Cigarette Pack Years	16 ± 16	15 ± 12	18 ± 18	NS	NS	NS
Total Cigarettes per day	19 ± 8	12 ± 8	17 ± 7	NS	0.003	0.006
Years Smoking	23 ± 11	24 ± 11	23 ± 10	NS	NS	NS
1 year avg. alcohol [drinks/mo]	47 ± 101	307 ± 180	23 ± 20	NS	0.000	0.000
3 year avg. alcohol [drinks/mo]	58 ± 108	307 ± 173	23 ± 20	NS	0.000	0.000
Lifetime avg. alcohol [drinks/mo]	57 ± 52	204 ± 131	26 ± 13	0.001	0.000	0.000
Years Drinking any alcohol	23 ± 12	28 ± 11	8 ± 14	0.000	NS	0.000
Beck Depression Inventory	11.6 ± 8.2	13.6 ± 8.7	5.7 ± 4.5	0.002	NS	0.000
STAI State	33.0 ± 9.8	43.3 ± 12.2	30.3 ± 6.2	NS	0.002	0.000
STAI Trait	39.0 ± 11.6	37.9 ± 12.6	34.3 ± 9.6	NS	NS	NS
*Cognitive domains (z-scores)*						
Cognitive Efficiency	−0.15 ± 0.40	−0.12 ± 0.54	−0.21 ± 0.50	NS	NS	NS
Executive Functioning	−0.12 ± 0.60	−0.34 ± 0.62	−0.64 ± 0.70	0.01	NS	NS
Processing Speed	−0.21 ± 0.50	−0.19 ± 0.64	−0.29 ± 0.51	NS	NS	NS
Visuospatial Skills	−0.08 ± 0.96	0.15 ± 0.93	0.19 ± 0.80	NS	NS	NS
Working Memory	−0.10 ± 0.47	−0.04 ± 0.70	0.14 ± 0.66	NS	NS	NS
Global Cognition	−0.02 ± 0.45	−0.02 ± 0.49	0.11 ± 0.45	NS	NS	NS
*Self-regulation*						
BIS-11 Total Impulsivity	67.3 ± 11.2	66.3 ± 10.5	65.8 ± 12.0	NS	NS	NS
BIS-11 Attention	17.3 ± 4.1	16.7 ± 4.0	16.0 ± 4.3	NS	NS	NS
BIS-11 Motor	24.4 ± 5.1	23.3 ± 5.1	24.5 ± 4.3	NS	NS	NS
BIS-11 Nonplanning	25.6 ± 4.8	26.3 ± 4.6	25.4 ± 5.3	NS	NS	NS
BART [adjusted avg pumps]	33.1 ± 12.3	25.7 ± 11.7	35.3 ± 14.8	NS	0.071	0.029
IGT [net total]	13.2 ± 35.6	15.9 ± 26.7	6.0 ± 27.8	NS	NS	NS
*Laboratory variables*						
MCV [fl]	89.7 ± 5.6	94.6 ± 6.4	93.1 ± 3.8	NS	0.008	NS
γ-GTP [U/l]	18.5 ± 10.8	59.8 ± 44.5	17.0 ± 4.5	NS	0.000	0.042
Anion Gap [mmol/L]	7.9 ± 1.7	8.6 ± 1.9	6.9 ± 1.8	NS	NS	0.006
Potassium [mmol/L]	4.0 ± 0.3	3.8 ± 0.3	4.3 ± 0.6	NS	NS	0.019
Blood Urea Nitrogen [mg/dL]	14.6 ± 4.4	13.3 ± 4.3	18.5 ± 4.8	NS	NS	0.012
Hemoglobin [g/dl]	13.3 ± 1.3	14.6 ± 1.3	14.8 ± 1.5	0.036	0.002	NS
Hematocrit [%]	38.9 ± 3.7	42.6 ± 4.1	43.2 ± 4.0	0.024	0.003	NS

**Table 2:** Substance use histories of the OD group.

Substance	%	Duration in yrs	Lifetime g/mo	Previous yr g/mo
Opiates	100	11 ± 7 (2–22)	16 ± 14 (0.04–43)	6 ± 13 (0.5–44; n=7)
Tobacco	100	23 ± 11 (7–45)	554 ± 242 (180–1200)^	not available
Alcohol*	90	23 ±12 (6–44)	57 ± 52 (1–94)	47 ± 101 (1–330)
Cocaine	71	5 ± 7 (1–25)	24 ± 52 (2–240)	3 ± 8 (0.25–32)
Methadone	62	2 ± 3 (1–10)	not available	not available
Marijuana	62	18 ± 13 (3–41)	17 ± 24 (2–90)	8 ± 23 (2–90)
Amphetamines	38	2 ± 3 (3–10)	4 ± 9 (0.5–38)	0.2 ± 1 (0.5–4; n=2)

Mean ± standard deviation; range in parentheses,

^ Cigarettes/mo,

* Quantities in alcoholic drinks per month; 1 drink defined as containing 13.6 g of ethanol.

**Table 3:** Cognitive domains formed and constituent measures.

Cognitive Domain	Constituent Measures
Executive functions	• Short Categories Test [75]
	• Stroop Test, color-word subtest [76]
	• Trail Making Test B [77]
	• Wisconsin Card Sorting Test-64 (WCST-64): Computer Version 2-Research Edition non-perseverative errors, perseverative errors, perseverative responses [78]
Visuospatial skills	• Wechsler Adult Intelligence Scale 3rd Edition (WAIS-III) Block Design [79]
Processing speed	• WAIS-III Digit Symbol [79]
	• WAIS-III Symbol Search [79]
	• Stroop, colour-word subtests [76]
	• Trail Making Test A [77]
Working memory	• WAIS-III Arithmetic [79]
	• WAIS-III Digit Span [79]
Global cognition	• The arithmetic average of z-scores for all of the individual cognitive domains
Cognitive efficiency	• The arithmetic average z-scores for tests that were timed, or where the time to complete the task influenced the score obtained
	• Stroop Test, colour-word subtest [76]
	• Trail Making Test A and B [77]
	• WAIS-III Arithmetic, Block Design, Digit Symbol, Symbol Search [79]

**Table 4:** Percent grey matter (GM), white matter (WM), and cerebrospinal fluid (CSF) contributions to the four volumes of interest (VOIs) (mean ± standard deviation), *WM or #GM tissue fraction differed in pairwise group comparisons (p<0.05).

VOI	Group	Number of smokers/non-smokers	WM Fraction	CSF Fraction	GM Fraction
ACC*	OD	21/0	36.0 ± 0.2	17.1 ± 2.3	46.1 ± 3.7
	ALC	28/0	34.7 ± 5.0	18.4 ± 3.4	45.9 ± 3.0
	CON	27/0	32.8 ± 3.8	19.2 ± 3.6	47.0 ± 2.6
DLPFC	OD	20/0	53.2 ± 7.4	6.3 ± 3.0	39.6 ± 5.4
	ALC	23/0	51.7 ± 6.8	7.0 ± 3.1	40.4 ± 4.6
	CON	27/0	51.3 ± 6.9	7.3 ± 3	40.5 ± 4.7
OFC ^#^	OD	14/0	58.8 ± 3.4	3.3 ± 1.2	37.1 ± 3.0
	ALC	12/9	55.6 ± 5.5	4.7 ± 2.3	38.9 ± 4.6
	CON	5/14	58.8 ± 6.1	4.9 ± 2.7	35.5 ± 4.3
POC	OD	18/0	29.2 ± 4.6	7.3 ± 2.0	62.7 ± 4.5
	ALC	29/0	27.5 ± 5.0	10.3 ± 5.4	61.3 ± 4.1
	CON	6/2	28.8 ± 5.2	9.5 ± 3.1	60.9 ± 4.4

**Table 5:** Region specific metabolite concentrations for OD, ALC, CON, (mean ± standard deviation) effect sizes (Cohen’s d), and group statistics (ANCOVA), Reported values are estimated mean and standard deviation in institutional units from a 3-group ANCOVA with age and tissue contribution as covariates as needed, NS: Not significant; Significant group effects in bold, * Significant pairwise group comparison after Bonferroni adjustment.

ROIs & Metabolites	Mean ± SD			Effect Sizes			Group Significance
	OD	ALC	CON	OD vs. CON	OD vs. ALC	ALC vs. CON	
DLPFC NAA	4.0 ± 0.6	4.7 ± 0.6	5.0 ± 0.6	1.59*	1.09*	0.5	**F(2,68)=14.82, p<0.001**
DLPFC CR	3.3 ± 0.6	4.3 ± 0.6	4.2 ± 0.6	1.64*	1.67*	0.03	**F(2,70)=19.22, p<0.001**
DLPFC CHO	1.0 ± 0.2	1.1 ± 0.2	1.0 ± 0.2	0.46	0.71*	0.25	NS
DLPFC MI	2.3 ± 0.7	3.0 ± 0.7	3.0 ± 0.7	0.94*	0.93*	0.01	**F(2, 69)=6.12, p=0.004**
DLPFC GLU	2.7 ± 0.5	3.0 ± 0.5	3.2 ± 0.5	1.00*	0.65	0.36	**F(2, 70)=5.74, p=0.005**
DLPFC GABA	1.4 ± 0.4	1.5 ± 0.4	1.5 ± 0.4	0.23	0.11	0.12	NS
ACC NAA	4.8 ± 0.8	4.8 ± 0.8	5.4 ± 0.8	0.78*	0.01	0.79*	**F(2,76)=5.22, p=0.009**
ACC CR	3.7 ± 0.7	3.8 ± 0.7	4.5 ± 0.7	1.18*	0.21	0.96*	**F(2,74)=12.46, p<0.001**
ACC CHO	1.2 ± 0.2	1.2 ± 0.2	1.3 ± 0.2	0.45	0.12	0.57	NS
ACC MI	2.3 ± 1.0	3.6 ± 1.0	3.4 ± 1.0	1.15*	1.31*	0.17	**F(2,74)=10.96, p<0.001**
ACC GLU	3.3 ± 0.9	3.4 ± 0.8	3.8 ± 0.9	0.59	0.15	0.44	NS
ACC GABA	1.5 ± 0.5	1.4 ± 0.5	1.6 ± 0.5	0.11	0.21	0.32	NS
OFC NAA	4.9 ± 1.0	4.7 ± 0.9	4.8 ± 0.9	−0.12	−0.24	−0.12	NS
OFC CR	3.6 ± 1.0	3.9 ± 1.0	3.7 ± 1.0	0.11	0.3	0.19	NS
OFC CHO	0.9 ± 0.2	1.0 ± 0.2	0.8 ± 0.2	−0.61	−0.54	−1.14*	**F(2,51)=5.84, p=0.005**
OFC GLU	2.6 ± 0.7	3.0 ± 0.7	2.4 ± 0.7	−0.24	−0.62	−0.86*	**F(2, 50)=3.76, p=0.031**
POC NAA	5.3 ± 0.8	5.1 ± 0.8	4.9 ± 0.8	−0.49	−0.25	−0.24	NS
POC CR	4.3 ± 0.6	4.3 ± 0.6	4.1 ± 0.6	−0.34	0.01	−0.33	NS
POC CHO	0.8 ± 0.1	0.8 ± 0.1	0.7 ± 0.1	−0.86	0.41	−0.45	NS
POC MI	2.9 ± 0.9	2.3 ± 0.9	2.8 ± 0.9	−0.14	−0.67	−0.53	NS
POC GLU	3.7 ± 0.6	3.6 ± 0.6	3.7 ± 0.6	0.09	−0.14	−0.23	NS
POC GABA	1.7 ± 0.3	1.5 ± 0.3	1.5 ± 0.3	−0.62	−0.48	0.13	NS
